# Terminal ileitis and cytotoxic lesion of corpus callosum as the presenting features of Multisystem inflammatory syndrome in children (MIS-C): a case report

**DOI:** 10.1186/s12887-022-03707-2

**Published:** 2023-01-11

**Authors:** Marzieh Davoodi, Gholamreza Pouladfar, Mohammad Rahim Kadivar, Alireza Dehghan, Aida Askarisarvestani, Seyedeh Sedigheh Hamzavi

**Affiliations:** 1 Student Research Committee, Shiraz University of Medical Sciences, Shiraz, Iran; 2 Professor Alborzi Clinical Microbiology Research Center, Shiraz University of Medical Sciences, Shiraz, Iran; 3 Division of Pediatric Infectious Diseases, Department of Pediatrics, School of Medicine, Shiraz University of Medical Sciences, Shiraz, Iran; 4 Medical Imaging Research Center, Shiraz University of Medical Sciences, Shiraz, Iran; 5 Division of Allergy and Clinical Immunology, Department of Pediatrics, School of Medicine, Shiraz University of Medical Sciences, Shiraz, Iran

**Keywords:** MIS-C, Cytotoxic lesions of the corpus callosum, Terminal ileitis, Seizure, Case report21-24

## Abstract

**Background:**

Multisystem inflammatory syndrome in children (MIS-C) is a post-viral inflammatory vasculopathy characterized by persistent fever, multiorgan dysfunction, significant laboratory markers of inflammation, lack of an alternative diagnosis, and prior SARS-CoV-2 infection or exposure in children and adolescents. The most common early symptoms include a prolonged fever, as well as dermatologic, mucocutaneous, and gastrointestinal symptoms such abdominal pain, vomiting, and diarrhea.

**Case presentation:**

We present a pediatric patient with multisystem inflammatory syndrome with the development of abdominal pain and seizure who was found to have a circumferential wall thickening of the terminal ileum and ileocecal junction in abdominal CT scan. The brain MRI of the patient showed cytotoxic lesions of the corpus callosum (CLOCC) which had hypersignal intensity with a few diffusion restrictions in the splenium of the corpus callosum.

**Conclusion:**

This case is being reported to raise awareness of MIS-C presenting characteristics. Given the rising number of MIS-C patients and a lack of understanding regarding early diagnostic clinical characteristics and therapy, further research into clinical presentations, treatment, and outcomes is urgently needed.

## Background

Multisystem inflammatory syndrome in children (MIS-C) is a severe and potentially lethal complication of Severe Acute Respiratory Syndrome Coronavirus-2 (SARS-CoV-2) infection that presents with various clinical manifestations [1]. Although the pathophysiology of MIS-C is not well understood, various speculations have been made regarding the immunopathogenesis of this syndrome; such hypotheses include SARS-CoV-2 acting as a superantigen and inducing a cytokine storm, as well as molecular mimicry [2]. This syndrome is characterized by fever in conjunction with clinical and laboratory evidence of multisystem inflammation that leads to cardiovascular, pulmonary, nervous, hematological, and gastrointestinal dysfunction in patients with recent SARS-CoV-2 infection [3].

Gastrointestinal symptoms including abdominal pain, vomiting, and diarrhea are common in patients with COVID-19 infection and MIS-C and may mimic appendicitis due to the involvement of the terminal ileum [4–6]. Many patients with terminal ileitis related to SARS-CoV-2 infection have been reported, but few studies have described terminal ileitis as the presenting manifestation of MIS-C.

In this article, we describe a child with MIS-C presenting with fever, clinical and radiologic evidence of terminal ileitis, and seizure who was referred to a tertiary hospital in Iran and improved with medical management.

## Case presentation

The patient was an 11-year-old child weighing 38 kg who was admitted to Namazi Teaching Hospital affiliated to Shiraz University of Medical Sciences, Southern Iran with the chief complaint of fever and chills lasting from three days before hospitalization. He had also been suffering from watery diarrhea, several episodes of vomiting, intermittent abdominal pain, and two episodes of abnormal spastic movements in his upper and lower extremities, accompanied by a decreased level of consciousness, each lasting about 10 min. He was a known case of mitral valve regurgitation and had been receiving Propranolol for this condition. The patient had close contact with a COVID-19 patient one month prior to the presentation, but he denied having any previous respiratory symptoms. The patient was not vaccinated against COVID-19 due to the lack of vaccines for pediatric patients in Iran at that time.

On arrival, he seemed ill and was conscious with mild respiratory distress. He had a heart rate of 134 beats per minute, a blood pressure of 98/42 mmHg, a respiratory rate of 40 respirations per minute, and an oral temperature of 38 degrees Celsius; however, he was not dehydrated. His arterial oxygen saturation was 95% on room air. In his physical examination meningeal signs were not detected. On abdominal examination, guarding and generalized tenderness with the greatest intensity in the right lower quadrant was detected. Other physical examinations, including evaluation of the nervous system were normal. Urine culture and urine analysis, blood culture, and stool culture were sent from the patient for evaluation of the source of infection, all of which came back negative. Since the patient did not have any meningeal signs, lumbar puncture was not performed for him and cerebrospinal fluid culture is not available. Based on the working diagnosis of systemic sepsis secondary to suspected appendicitis and COVID-19, the patient was isolated and ceftriaxone (100 mg/kg/day), metronidazole (30–40 mg/kg/day divided into three doses), and phenytoin (loading dose, 20 mg/kg and then 5 mg/kg/day) were prescribed intravenously. His initial abdominopelvic ultrasonography revealed circumferential edematous wall thickening in the terminal ileum and the ileocecal valve with multiple varying size hypoechoic lymph nodes in the right parailiac area, the largest of which measured 27 × 16 mm. The COVID-19 evaluation included Polymerase Chain Reaction (PCR) of the nasopharyngeal sample for SARS-CoV-2, COVID-19 serology, and inflammatory markers for MIS-C (Table 1). On the third day of admission, he developed a maculopapular rash all over his body and redness in his oral mucosa and lips. Table 1 shows the patient's laboratory data during hospitalization.

**Table 1:** The results of the patient’s lab data during his hospitalization

Lab Data	first day of admission	Second day of admission	Third day of admission	Seventh day of admission
**WBC, count/mm**^**3**^	**3500**	**4900**	**20,800**	
**Hemoglobin, g/dL**	**11.1**	**10.8**	**11.4**	
**MCV, fL**	**70.3**		**73.1**	
**Platelet /mm**^**3**^	**105,000**	**107,000**	**152,000**	
**Prothrombin time, sec**	**16.7**	**15**	**13.8**	
**INR, index**	**1.21**	**1.09**	**1**	
**PTT, sec**	**45.3**	**43.9**	**36.1**	
**Blood sugar, mg/dL**	**123**	**142**		
**BUN, mg/dL**	**9**	**9**	**14**	
**Cr, mg/dL**	**0.7**	**0.7**	**0.7**	
**Na, mEq/L**	**137**	**136**		
**K, mEq/L**	**2.8**	**4.9**		
**AST, IU/L**	**140**		**27**	
**ALT, IU/L**	**120**		**39**	
**ALK.P, IU/L**	**569**		**299**	
**Total Bilirubin, mg/dL**	**0.6**		**0.6**	
**Direct Bilirubin, mg/dL**	**0.3**		**0.3**	
**Amylase, U/L**		**62**		
**Total protein, g/dL**	**5.8**		**7.3**	
**Albumin, g/dL**	**3.3**		**3**	
**LDH, IU/L**	**674**		**549**	
**ESR, mm/h**	**72**			**66**
**CPK, IU/L**	**112**		**30**	
**Wright**	**Negative**		**Negative**	
**CRP, mg/L**	** > 150**		** > 150**	**13**
**Ferritin, ng/mL**		**1514.6**		
**Troponin I rapid test**	**16.1**			
**D-dimer, mg/L**		**7.15**		
**Blood culture**				**Negative**
**Urine culture**				**Negative**
**Stool OB-OP**		**Negative**	**Negative**	
**SARS-CoV-2 PCR**	**Negative**		**Negative**	
**SARS-CoV-2 IgG** **(Positive > 1.1)**		**6.39**		
**SARS-CoV-2 IgM** **(Positive > 1.1)**		**0.57**		

*INR* International normalized ratio, *MCV*, Mean corpuscular volume, *PTT*, Partial prothrombin time, *BUN* Blood urea nitrogen, *Cr* Creatinine, *Na* Sodium, *K* Potassium, *AST* Aspartate transaminase, *ALT* Alanine transaminase, *ALK.P* Alkaline phosphatase, *LDH* Lactate dehydrogenase, *ESR* Erythrocyte sedimentation rate, *CPK* Creatine phosphokinase, *CRP* C-reactive protein, *OB-OP* Occult blood, Ova, parasite

**Fig. 1:**
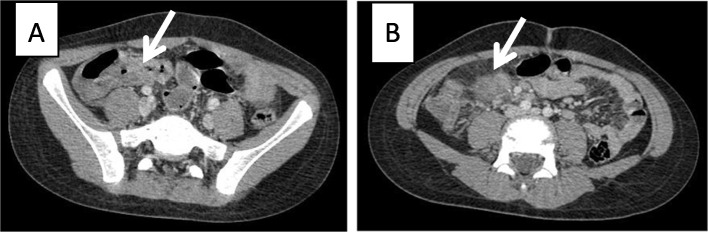
Axial contrast-enhanced CT images show circumferential wall thickening of the terminal ileum and ileocecal junction (thick arrow in A) and prominent inflammatory mesenteric lymph nodes with adjacent fat stranding (thick arrow in B)

**Fig. 2:**
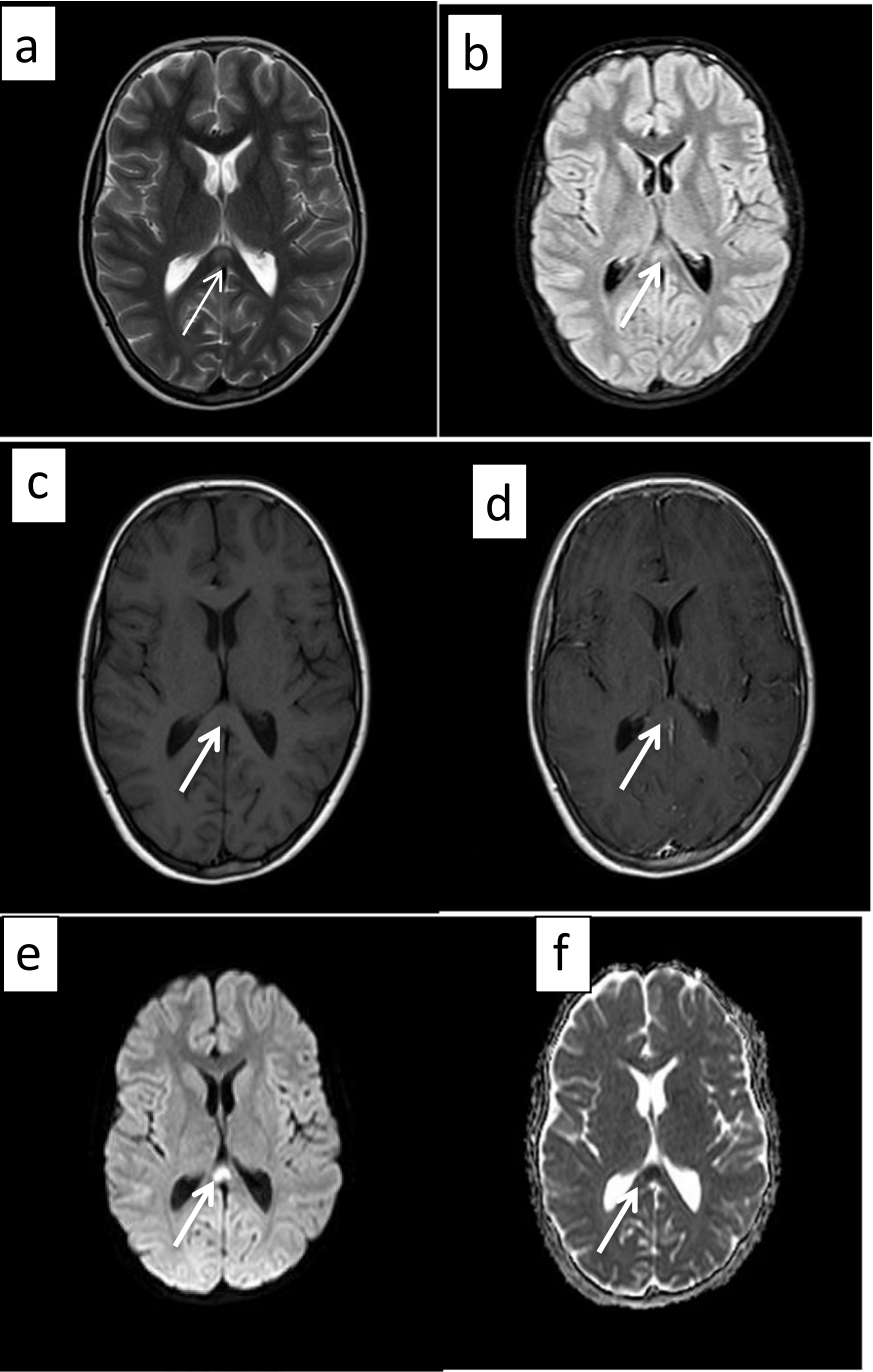
CLOCC in an 11-year-old boy with MIS-C. Brain MRI shows a lesion in the splenium of the corpus callosum (arrows) which is hypersignal on T2W (**a**) and FLAIR (**b**) sequences and was hyposignal on pre-contrast T1W sequence (**c**). There is no abnormal enhancement after the administration of the contrast agent (**d**). There is also increased signal intensity on DWI sequences at b 1000 s/mm2 (**e**) associated with loss of signal on the apparent diffusion coefficient map (f). These findings represent a focus of cytotoxic edema in the splenium of the corpus callosum

**Fig. 3:**
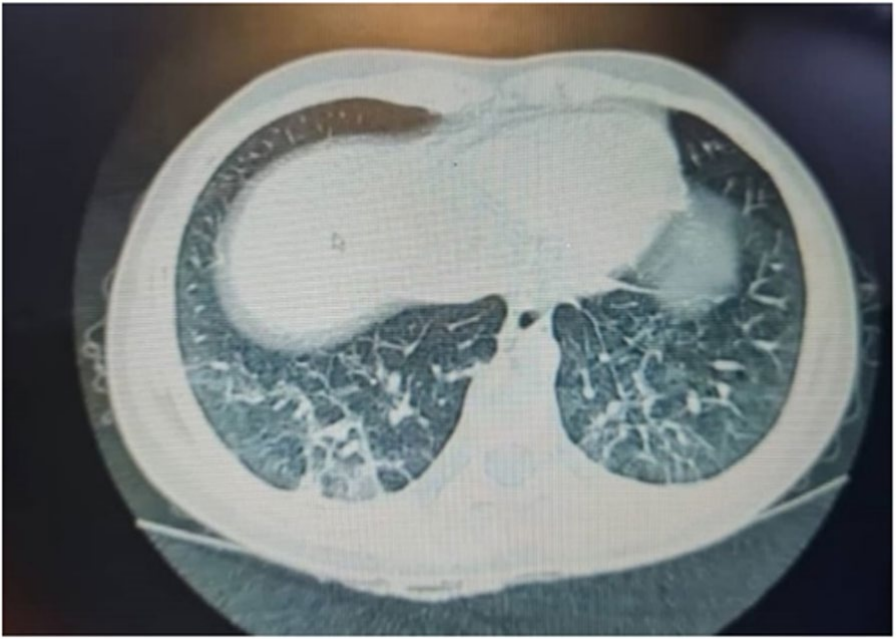
Ground glass opacities (GGO) in the lower parts of both lungs

The result of SARS-CoV-2 PCR on the patient’s nasopharyngeal sample came back negative, but the serology for Anti-SARS-CoV-2 IgG was positive, which shows a comparatively remote infection with the virus. Laboratory investigations revealed leukopenia, microcytic anemia, thrombocytopenia, hypoalbuminemia, and elevated levels of liver enzymes, C-Reactive Protein (CRP), Erythrocyte Sedimentation Rate (ESR), Lactate Dehydrogenase (LDH), and Ferritin. The patient underwent an ultrasound and then a computed tomography (CT) scan of the abdomen, which showed thickening of the terminal ileum and the ileocecal valve and lymphadenopathy in the right iliac region (Fig. 1). The Electroencephalogram (EEG) of the patient showed some paroxysmal generalized epileptic discharges. The brain MRI showed cytotoxic lesions in the corpus callosum with a hypersignal intensity and a few diffusion restrictions in the splenium of the corpus callosum (SCC) in T2 and FLAIR views (Fig. 2). The lung CT scan showed ground-glass opacities (GGO) in the lower parts of both lungs, especially on the right side (Fig. 3). Echocardiography of the patient was normal except for a moderate mitral valve regurgitation.

Considering the diagnosis of MIS-C, intravenous Methyl Prednisolone (2 mg/kg/day) and Intravenous Immunoglobulin (IVIG) infusion (2 g/kg/day) were started. During the 10 days of hospitalization, fever, diarrhea, and abdominal pain gradually improved, and he was discharged with good general condition. He was prescribed oral Prednisolone (1 mg/kg/day) and Phenytoin (5 mg/kg/day) after discharge from the hospital. Prednisolone was tapered and eventually discontinued after two weeks without any recurrence of symptoms during the follow-up period.

The patient's electroencephalogram was repeated, which was normal; therefore, phenytoin was discontinued since seizures did not recur in the one-month neurologic follow-up. The patient was well in the follow-up period, but no follow-up imaging was done for the patient.

## Discussion and conclusion

We presented an 11-year-old boy with MIS-C, who presented with fever and multisystem involvement of the gastrointestinal tract (diarrhea, abdominal pain, terminal ileitis, and elevated liver enzymes), the nervous system (seizure, lesions in SCC), the respiratory system (GGO in the lungs), and the skin (maculopapular rash), with laboratory evidence of inflammation (lymphocytopenia, hypoalbuminemia, and elevated levels of CRP, ESR, Ferritin, and LDH), no other apparent microbial cause of inflammation, and evidence of SARS-CoV-2 infection (positive serology for COVID-19). Both COVID-19 and MIS-C have gastrointestinal manifestations, but terminal ileitis has been reported in a few children with COVID-19 and fewer patients with MIS-C.

Terminal ileitis is the inflammation of the end-portion of the ileum that may present acutely as abdominal pain, mainly in the right lower quadrant with or without diarrhea, chronic bowel obstruction symptoms, and gastrointestinal bleeding that could mimic acute appendicitis [7]. TI usually occurs in association with a wide range of etiologies, including inflammatory bowel diseases (Crohn's disease and, to a lesser frequency, ulcerative colitis), taking nonsteroidal anti-inflammatory drugs, intestinal ischemia, eosinophilic enteritis, neoplasms (lymphoma), vasculitis, spondyloarthropathy, lymphoid hyperplasia, and infectious agents. Bacterial pathogens such as *Mycobacterium tuberculosis*, *Yersinia*, *Salmonella*, and *Clostridium difficile* have been reported as infectious agents causing TI [8–11]. Viral pathogens such as *Cytomegalovirus* can also cause TI [12]. In two case reports, TI was recently reported in two children (11and 12 years old) with SARS-CoV-2 infection who presented with acute abdomen [13, 14].

Only a few cases of TI in children with MIS-C have been reported. In a short report from a single center in the UK, eight children with COVID-19 and symptoms of atypical appendicitis whose imaging studies confirmed TI were reported. Three patients developed Systemic Inflammatory Response Syndrome; two of them were initially planned for appendectomy, but the decisions for the surgical intervention were subsequently overturned due to hemodynamic instability or a positive SARS-CoV-2 PCR. Ultimately, all children improved without any surgical interventions [15]. In a single-center study from the USA, 34 out of 35 (97%) children with MIS-C had gastrointestinal symptoms, and 19 out of 35 had moderate to severe abdominal symptoms that warranted radiographic imaging. In four out of seven patients for whom CT scan of the abdomen were done, terminal ileitis was detected. In two out of fourteen patients for whom abdominal ultrasonography was performed, mild ileal thickening was reported. In one patient with bowel obstruction symptoms who underwent ileocolic resection, there was a 6 cm mass in the ileocolic pedicle and a 3 cm span of granular, thickened terminal ileal mucosa. All other patients improved with medical therapy, including Corticosteroids, Aspirin, Infliximab, and at least one dose of IVIG [3].

In a study by Yack-Corrales et al. 1010 pediatric patients with COVID-19 or MIS-C were evaluated for acute abdomen and appendicitis. Even though 34 patients were diagnosed with appendicitis, no case of terminal ileitis was found in that large cohort; therefore, such diagnoses are extremely rare [4].

In another cohort study conducted on 32 children with MIS-C and left ventricular dysfunction or cardiogenic shock admitted to 15 hospitals in France and Switzerland, two patients underwent emergency laparotomy for suspected appendicitis; however, they were finally diagnosed with mesenteric lymphadenitis [16]. In another short report from South Africa, two of 23 children with MIS-C underwent operation for suspected appendicitis. The etiology of acute abdomen was not explained in the report [17].

Seizure was an indicative symptom of neurologic involvement in this patient. There was hypersignal intensity with a few diffusion restrictions in the splenium of the corpus callosum (SCC) detected in his brain MRI. There is a wide range of etiologies leading to lesions in SCC, including callosal malformations, disorders of myelination (X-linked adrenoleukodystrophy and hereditary Krabbe's disease), tumors, hypoglycemia, ischemia due to hypoxia, epilepsy itself, diffuse axonal injury secondary to trauma, Marchiafava–Bignami disease secondary to chronic alcohol abuse and vitamin B12 deficiency, post-shunt decompression in chronic hydrocephalus, certain antiepileptic drugs, encephalopathy due to Hemolytic Uremic Syndrome, infectious agents (Influenza virus, Rotavirus, Mumps virus, *Escherichia coli*, Adenovirus, *Aspergillus*, *Mycobacterium tuberculosis*), AIDS dementia complex, Mild Encephalitis/Encephalopathy with Reversible isolated SCC lesion (MERS), Posterior Reversible Encephalopathy Syndrome (PRES), and Multiple Sclerosis (MS) [18–20]. The inflammation of brain tissue or vessels is the most probable etiology of lesions in SCC in this patient. We postulate that the cytotoxic lesion of the corpus callosum in the index case, was secondary to the systemic inflammation from SARS-CoV-2 infection, resulting in Multisystem Inflammatory Syndrome in Children.

Appleberry et al. reported a case with status epilepticus in the context of MIS-C with post-ictal cerebral edema. The patient received pulse steroids and IVIG which resulted in improvement of his condition; however, long-term neurologic deficits such as dysphagia and developmental regression persisted [21]. Fortunately, the neurologic manifestations of our case were resolved after proper treatment with pulse steroids and IVIG.

The occurrence of seizure secondary to the cytotoxic lesion of the corpus callosum and severe abdominal tenderness secondary to TI as the presenting manifestations in this patient urged us to report him to convey an essential best practice message. Physicians should consider the possibility of cytotoxic lesion of the corpus callosum and TI in MIS-C patients with seizure and severe acute abdomen symptoms, perform timely imaging studies, and start proper medical treatment as soon as possible to avoid unnecessary surgical interventions and complications.

## Limitations

It would be better if the patient repeated his brain MRI and abdominopelvic sonography to evaluate the reversibility of such findings, but follow-up was achieved by clinical findings and EEG alone. CSF examination was also not performed for the patient since he did not have any meningeal signs.

## Data Availability

Not applicable.

## Abbreviations

MIS-C: Multisystem inflammatory syndrome in children
CLOCC: Cytotoxic lesions of the corpus callosum
SCC: Splenium of the corpus calusom
GGO: Ground glass opacity
IVIG: Intravenous immunoglobulin
TI: Terminal ileitis
